# Microwave-Assisted Synthesis of Novel Ni_3_S_2_/Ce_2_O_2_S 2D Hexagonal Nanoflakes for High-Performance Asymmetric Supercapacitors

**DOI:** 10.2147/NSA.S562196

**Published:** 2025-12-24

**Authors:** Muhammad Saleem Akhtar, Tomasz Wejrzanowski, Gabriela Komorowska, Emilia Choinska, Magdalena Laskowska, Zaeem Ur Rehman, Marcin Łapiński

**Affiliations:** 1 Faculty of Materials Science and Engineering, Warsaw University of Technology, Warsaw, Poland; 2 Institute of Nuclear Physics Polish Academy of Sciences, Krakow, Poland; 3 Faculty of Electronics, Telecommunications and Informatics, Gdańsk University of Technology, Gdańsk, Poland; 4 Faculty of Applied Physics and Mathematics, Institute of Nanotechnology and Materials Engineering, Gdansk University of Technology, Gdańsk, Poland

**Keywords:** 2D hexagonal nanoflakes, microwave-assisted hydrothermal synthesis, specific capacitance, energy density, asymmetric supercapacitor

## Abstract

**Introduction:**

High-energy-density supercapacitors require advanced electrode materials with superior pseudocapacitive behavior and stability. This study focuses on the design and development of binder-free pseudocapacitive electrodes composed of two-dimensional (2D) hexagonal nickel/cerium sulfide nanoflakes, which are directly synthesized on nickel foam. The aim was to achieve enhanced electrochemical performance through novel 2D nanoarchitectures and improved charge transfer dynamics.

**Methods:**

The nickel/cerium sulfide nanoflakes were fabricated via a microwave-assisted hydrothermal synthesis. Structural and morphological characteristics were analyzed using X-ray diffraction (XRD), Raman spectroscopy, scanning electron microscopy (SEM), and X-ray photoelectron spectroscopy (XPS). Electrochemical properties were evaluated through cyclic voltammetry, galvanostatic charge–discharge, and electrochemical impedance spectroscopy in both half-cell and asymmetric supercapacitor (ASC) configurations.

**Results and Discussion:**

The synthesized electrode demonstrated a high specific capacitance of 5286 F/g, an energy density of 222.09 Wh/kg, and a power density of 687.19 W/kg at 2.5 A/g in the half-cell system. The ASC device, utilizing nickel/cerium sulfide nanoflakes as the positive electrode and graphene nanoplatelets (GNPs)@Ni foam as the negative electrode, achieved an energy density of 77.51 Wh/kg and a power density of 797.25 W/kg at 1 A/g. The ASC also demonstrated excellent cyclic durability, retaining 84% of its capacitance after 10,000 cycles.

**Conclusion:**

The in situ-grown 2D hexagonal nickel/cerium sulfide nanoflakes exhibit outstanding pseudocapacitive behavior and electrochemical stability, underscoring their strong potential for next-generation high-performance asymmetric supercapacitors.

## Introduction

Electrochemical energy storage devices are crucial for designing modern electric hybrid vehicles and portable electronic gadgets. Numerous energy storage technologies have been established, including zinc-ion batteries (ZIBs), potassium-ion batteries (PIBs), sodium-ion batteries (SIBs), lithium-ion batteries (LIBs), and supercapacitors (SCs). Among these, supercapacitors have gained significant consideration owing to superior power density, extended operational period, and fast charge/discharge aptitude, confirming them as a promising option for energy storage systems.^1^,^2^ A supercapacitor device typically comprises a separator, an electrolyte, two electrodes designated as the anode and cathode, and a sealing/casing. The electrode material utilized in the SCs is the most critical component of the device’s performance. Electrode materials are classed into three primary categories depending on the charge accumulation process: electric double layer type (EDLC), pseudocapacitive type, and hybrid materials. EDLC-type materials exhibit charge storage by forming two layers at the electrode/electrolyte boundary, whereas pseudocapacitive materials retain charges through reversible Faradic redox reactions. Hybrid electrode materials combine charge storage mechanisms, incorporating the electrostatic type from EDLC and the Faradic type from pseudocapacitive materials.^3^,^4^ Furthermore, based on the negative and positive electrode materials, SCs are categorized according to the device’s configuration, specifically symmetric and asymmetric type devices. In asymmetric supercapacitor-type devices, pseudo-capacitive materials are utilized as positive electrodes, while EDLC-type materials serve as negative electrode materials.^5^ By leveraging the properties of these two dissimilar materials, asymmetric supercapacitors (ASCs) can operate within a wide range of potential windows, exhibiting enhanced power density compared to pseudo-capacitive type materials and increased energy density relative to EDLC-type materials. ASCs have the potential to provide significantly higher power densities than batteries and fuel cells, rendering them more suitable for addressing escalating energy demands. Consequently, ASCs demonstrate promising prospects as efficient energy storage devices. Asymmetric supercapacitor (ASC) performance predominantly depends on the pseudocapacitive electrode, facilitating expeditious and changeable redox responses at the surface to transfer charge through ion intercalation or adsorption proximal to the electrode interface. In comparison to electrical double-layer capacitors, pseudo-capacitors exhibit comparable rate competencies and almost the same electrochemical properties, as demonstrated through electrochemical techniques.^6^

Scientists have recently explored various pseudocapacitive materials, incorporating conducting polymers,^7^ transition metal hydroxides,^8–11^ oxides,^12^ and sulfides,^4^,^13^ Metallic sulfides have drawn considerable admiration among these materials owing to their favorable redox reactions and tunable morphologies.^14^,^15^ Nickel-based sulfides, in particular, have been of interest owing to their rich redox capability, multiple oxidation states of nickel Ni^2+^/Ni^3+^ and sulfur S^2+^/S^4+^, higher capacitance, natural abundance, environmental compatibility, and superior conductivity.^2^,^16^,^17^ Significant efforts have been made to meet energy storage demands; however, nickel-based compounds alone have not achieved the desired performance.^18^,^19^ An alternative approach to enhance storage performance involves the preparation of nickel sulfide composites with other elements. Notably, bimetallic nickel cobalt sulfide has demonstrated exceptional performance.^20^,^21^ To date, nickel-cobalt-based compounds have been the most extensively studied pseudocapacitive electrode materials among the transition metal family.^9^,^21–30^ However, using cobalt in energy storage devices significantly increases their cost. Furthermore, factors such as mining, expenses, availability, and geopolitical considerations render reliance on cobalt-based compounds problematic.^31^ Researchers are actively seeking alternative elements that do not compromise the functioning of energy storage devices. Alternatively, among the family of rare earth metals, cerium is the most ample. Recently, cerium has garnered considerable attention and is being investigated for electrode materials in asymmetric supercapacitors (ASC) because of its low price, eco-friendly, and reversible oxidation states Ce^3+^/Ce^4+^.^32–35^ For example, Padmanathan et al reported the hydrothermal synthesis of carbon-supported CeO_2_ nanorods in 12 hours of processing time, followed by a 2-hour carbonization process, resulting in a capacitance of 644 F g^−1^ at a current density of 0.5 A g^−1^.^36^ Xu et al have reported a solvothermal method with a processing time of 6 hours for the synthesis of heterostructure nanosheet arrays of Zn/CoS@CeO_2_/NF, and demonstrated at a current density of 1 A g^−1^ a capacitance of 1638 F g^−1^. Furthermore, they fabricated an asymmetric supercapacitor (ASC) device utilizing Zn/CoS@CeO_2_/NF//AC. The resultant ASC exhibited energy density of 42.4 Wh/kg and electrochemical stability of 91.1% after 8000 cycles.^37^ Anit et al have reported two-dimensional CeO_2_ nanoflakes prepared in 8 hours of processing time by the solvothermal route, followed by 2 hours post-annealing of powder, and reported a capacitance of 1801 F g^−1^ at a current density of 1 A g^−1^ of electrode prepared by two-dimensional CeO_2_ nanoflakes in PVDF binder and coated on nickel foam. They achieved a maximum energy density of 57.6 Wh/kg and a power density of 771 W/kg in the fabricated ASC device based on CeO2//AC, with capacitance retention of approximately 83% after 1000 cycles.^38^ Amit et al have reported a composite of nanostructured CeO_2_/NiV–LDH, prepared via a conventional hydrothermal route over 12 hours, and observed a capacitance of 2378 F g^−1^ at a current density of 2 A g^−1^. Moreover, they fabricated an ASC device utilizing CeO_2_/NiV–LDH//Bi_2_O_3_ as positive and negative electrodes, respectively, and demonstrated exceptional electrochemical performance of the ASC device, with an energy density of 62.5 Wh/kg at a power density of 1595.2 W/kg at a current density of 2 A g^−1^.^39^ To the best of our knowledge, composites based on nickel/cerium sulfide have not been employed in ASCs as binder-free pseudocapacitive electrodes so far.

Furthermore, the electrochemical properties of Faradaic materials can be enhanced via a binder-free approach, as it preserves all active sites and provides an increased surface area without compromising the performance of the electroactive materials. Additionally, the binder-free approach is more efficient and cost-effective due to the elimination of conductive substances and binders.^13^,^20^ Binder-free electrodes can be fabricated using various methods; the most commonly reported is the hydrothermal synthesis route, which is environmentally friendly and allows for control over nanomaterial architectures and high-yield output. However, a limitation of the conventional hydrothermal method is its lengthy processing time, typically requiring several hours. Alternatively, the microwave-assisted hydrothermal (MW-HT) synthesis method exemplifies the principles of green chemistry by offering a sustainable approach to the synthesis of advanced materials. This technique reduces waste and enhances atom economy while simultaneously improving product yield. The utilization of water as a benign solvent eliminates the need for hazardous organic solvents, thereby promoting safer chemical processes. The shortened reaction times facilitated by microwave irradiation enhance energy efficiency and reduce costs, while the mild operating conditions mitigate the risks associated with conventional hydrothermal techniques. MW-HT obviates unnecessary derivatization steps and favors the use of catalysts over stoichiometric reagents, thereby conserving chemical resources. Its adaptability enables the use of renewable feedstock and real-time reaction monitoring, thereby preventing pollution. MW-HT facilitates the design of safer, recyclable products, aligning with the principles of green chemistry. These advantages render MW-HT a safer, cost-effective, and environmentally benign alternative for synthesis.^40–43^ Therefore, in this study, the microwave-assisted hydrothermal synthesis method is selected due to its versatility, capacity for morphological control, uniform heating, rapid processing, and cost-effectiveness.

Inspired by the promising redox properties of nickel and cerium, a binder-free approach, and a rapid microwave-assisted synthesis route, herein we, for the first time, report in situ grown 2D hexagonal nanoflakes Ni_3_S_2_/Ce_2_O_2_S on Ni foam. Two-dimensional (2D) nanoflakes synthesized on a nickel foam electrode exhibited exceptional electrochemical performance when evaluated in a half-cell configuration, primarily due to their distinctive 2D morphology. The electrode delivered a high specific capacitance of 5286 F/g, along with an energy density of approximately 222.09 Wh/kg and a power density of 687.19 W/kg at a current density of 2.5 A/g. These impressive electrochemical characteristics, including high capacitance and excellent rate capability, demonstrate the strong potential of this material for supercapacitor applications. For full-cell assembly, the 2D nanoflakes@Ni foam was used as the positive electrode, paired with a graphene nanoplatelet (GNP)@Ni foam electrode as the negative. The resulting asymmetric supercapacitor achieved an energy density of 77.51 Wh/kg and a power density of 797.25 W/kg at a current density of 1 A/g. Moreover, the device demonstrated outstanding cycling stability, retaining approximately 84% of its initial capacitance after 10,000 charge–discharge cycles, highlighting its long-term reliability and suitability for practical energy storage applications.

## Experimental

The following chemicals were used as supplied by their respective suppliers: potassium hydroxide (KOH) from Warchem, ammonium fluoride (NH_4_F) from Chempur, sodium sulfide (Na_2_S.9H_2_O) and cerium(III) nitrate hexahydrate (Ce(NO_3_)_3_.6H_2_O) from Sigma Aldrich, and nickel(II) nitrate hexahydrate (Ni(NO_3_)_2_.6H_2_O) and urea (CO(NH_2_)_2_) from Chempur. The Shanghai Tankii Alloy Material Limited in China provided a nickel foam substrate measuring 0.5 mm thick and featuring 90 pores per inch.

### Synthesis of Materials

**Figure 1:**
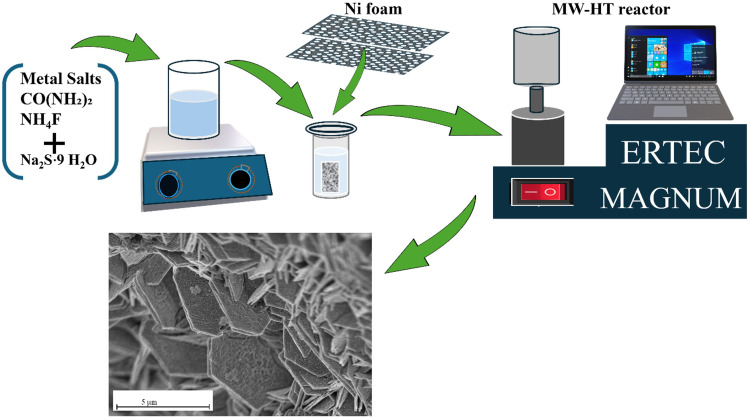
Synthesis procedure for 2D nanoflakes @ Ni foam.

The in situ growth of 2D hexagonal nanoflakes was accomplished through the following procedure. Initially, nickel foam supports (used as current collectors) with dimensions of 2 × 4 cm^2^ were cleaned with acetone and distilled water to remove organic moieties. Subsequently, the supports underwent acid treatment for 15 minutes in 37% hydrochloric acid in an ultrasound bath to create additional pores and increase the roughness of the Ni foam, thereby offering numerous active sites for the in situ synthesis of the desired electroactive product. Ni foam pieces were then rinsed in water to eliminate acid residue and overnight dried using a laboratory drier at 50°C. A solution containing metal salts, CO(NH_2_)_2_ as a hydrolysis agent, and ammonium fluoride, serving as a structure-directing mediator, was prepared in 40 mL of distilled water. Stoichiometric quantities of 2.6 mmol of nickel nitrate, 2.6 mmol of cerium nitrate, 1 mmol of NH_4_F, and 4 mmol of CO(NH_2_)_2_ were dissolved using the magnetic mixing process over 20 minutes. A separate solution was prepared in 20 mL of distilled water containing 15 mmol sodium sulfide as the sulfur source. This second solution was added dropwise to the previously prepared solution. The mixed solutions were then stirred for a further 15 minutes. After preparing the final reaction precursor solution, it was transported to the Teflon reaction vessel, where two pieces of Ni foam were submerged in the solution container. Subsequently, this Teflon vessel containing the current collector and reaction media was hermetically sealed in the microwave-assisted hydrothermal reactor (MW-HT) ERTEC MAGNUM II, Poland. The MW-HT reactor was programmed to maintain an average reaction temperature of 150°C for 4 hours, utilizing microwave irradiation as the heating source. Subsequently, the Ni foam, covered with 2D hexagonal nanoflakes, was rinsed several times with distilled water and then subjected to drying overnight at 50°C under vacuum conditions. The mass of loaded hexagonal nanoflakes on 1 × 1 cm^2^ Ni foam was estimated to be approximately 2 ± 0.2 mg/cm^2^. The average loading mass of electroactive material was calculated by determining the difference in weight between bare Ni foam and after the growth of 2D nanoflakes. The diagram of the synthesis procedure is shown in Figure 1.

### Materials Characterization

Two-dimensional nanoflakes directly synthesized on nickel foam were analyzed using various physicochemical techniques. Rigaku MiniFlex II (Japan) X-ray diffractometer was used to acquire X-ray diffraction (XRD) data. An inVia Raman microscope (Renishaw, Wotton-under-Edge, UK) equipped with a 633 nm He-Ne laser, a 1200 lines/mm grating, and a 100× objective lens was used for the Raman measurements. Using two accumulations and a 15-second exposure duration each scan, the spectra were acquired in static mode throughout a Raman shift range of 100 to 1000 cm^−^^1^. With the aid of an Autosorb iQ (Quantachrome Instruments, Boynton Beach, FL, USA), the nitrogen adsorption-desorption isotherms of the produced 2D nanoflakes on nickel foam were measured at 77.4 K. Prior to analysis, the material was degassed under vacuum for seven hours at 150°C. The specific surface area of the 2D nanoflakes produced directly on nickel foam was calculated using the Brunauer–Emmett–Teller (BET) equation, and the relative pressure range was chosen based on Rouquerol’s criterion.^44^ The Barrett–Joyner–Halenda (BJH) technique was used to calculate the pore size distribution (PSD) and pore volume from the desorption branch. ASIQWin 5.21 software was used for all computations. The morphology and elemental composition of the synthesized samples were analyzed using a Hitachi SU8000 scanning electron microscope (Japan) equipped with an energy-dispersive X-ray spectroscopy (EDX) detector. For a more detailed assessment of chemical composition, X-ray photoelectron spectroscopy (XPS) was employed. XPS measurements were conducted using a multi-chamber ultrahigh vacuum system (Omicron NanoTechnology) at ambient temperature, maintaining a base pressure below 1.1 × 10^−^^9^ mBar. Photoelectron excitation was achieved with an Mg Kα X-ray source operating at 15 keV and 300 W. The emitted photoelectrons were analyzed using an Omicron Argus hemispherical electron analyzer with a 128-channel detector. Measurements were performed in constant analyzer energy (CAE) mode with a pass energy of 40 eV. Binding energy calibration was carried out by referencing the C1s peak at 285.0 eV. The XPS spectra were processed utilizing Shirley background subtraction and fitted with Gaussian-Lorentzian (GL) curves using CasaXPS software.

### Electrochemical Measurements

All electrochemical investigations were conducted at room temperature using a Gamry potentiostat, Reference 3000 (Gamry Instruments USA).

#### Three-Electrode Setup

Without the use of binders or further processing, two-dimensional nanoflakes grown directly on nickel foam (1 × 1 cm^2^), utilized as the working electrode. In a three-electrode configuration, galvanostatic charge-discharge measurements and cyclic voltammetry were employed to assess the electrochemical performance. The electrolyte was a 2 M KOH aqueous solution, and the reference electrode was an Ag/AgCl electrode (in 3 M KCl), while the counter electrode was platinum foil. The capacitance value of the fabricated binder-free electrode could be estimated from Galvanostatic charge-discharge data following equation (1).^10^

(1) $${C_{sp}} = {{I \times \Delta t} \over {m \times \Delta V}}$$

where “C_sp_ is the capacitance in F g^−1^, m is the mass loaded on the current collector, $\Delta t$ is the discharge time and $\Delta V$ is the potential window”.

#### Two-Electrode Setup

The electrochemical performance of the fabricated asymmetric device was assessed using a two-electrode setup. The positive electrode consisted of two-dimensional hexagonal nanoflakes grown on nickel foam, while the negative electrode was formed by coating graphene nanopellets (GNPs) onto nickel foam. In detail, the Institute of Carbon Technologies in Toruń, Poland, supplied graphene nano pellets with specific characteristics (3 nm average sheet thickness, 1.5 μm diameter, and 800 m^2^/g specific surface area), which were utilized as received without further examination. To create the negative electrode slurry, 90 wt% of GNPs were combined with 10 wt% of polyvinylidene fluoride (PVDF) binder (Sigma Aldrich), using a minimal quantity of N-methyl-2-pyrrolidinone (NMP) as the solvent. The mixture was blended using a centrifugation mixer (THINKY ARV-930TWIN) for 10 minutes at 200 rpm. After preparing the slurry, it was applied via drop-casting onto a pre-cleaned Ni foam measuring 1 cm × 1 cm. The coated Ni foam was then dried overnight under a vacuum at 60°C. The mass ratio for the fabrication of the negative electrode was balanced using the equation to attain the optimal performance of the fabricated asymmetric device.^23^

(2) $${{{m_ + }} \over {{m_ - }}} = {{{C_ - }\Delta {V_ - }} \over {{C_ + }\Delta {V_ + }}}$$

In the above formula ∆V_±_, m_±_, and C_±_ are the positive and negative electrodes’ potential windows, mass of active material, and capacitance. The as-designed asymmetric supercapacitor’s energy density (ED) and power density (PD) could be evaluated by the following equations.^45^

(3) $$ED = {1 \over 2}C\Delta {V^2}$$

(4) $$PD = {{ED} \over {\Delta t}}$$

## Results and Discussion

### Materials Characterization

**Figure 2:**
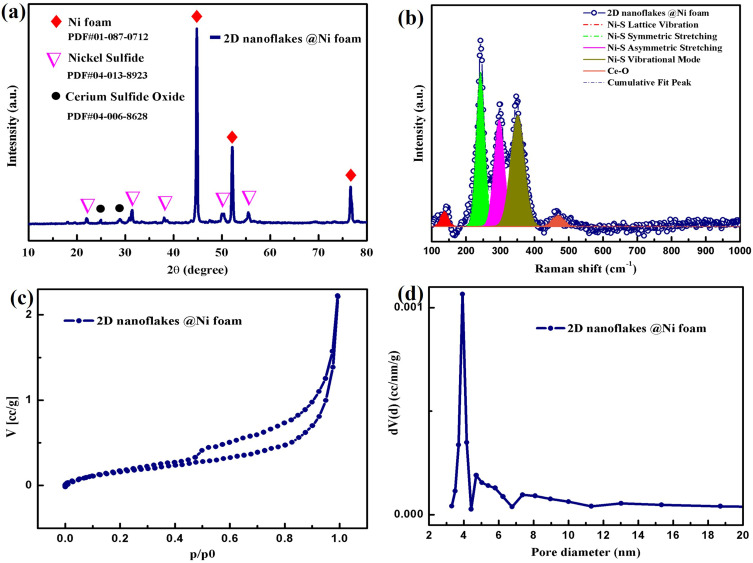
(**a**) XRD pattern of synthesized 2D nanoflakes @ Ni foam (**b**) Raman spectra of 2D nanoflakes @ Ni foam (**c**) N2 adsorption–desorption curves (**d**) pore size distribution of 2D nanoflakes @ Ni foam.

The phase composition and crystal structure of the 2D hexagonal nanoflakes produced directly on Ni foam were examined using X-ray diffraction (XRD) analysis. As illustrated in Figure 2a, the (101), (110), (021), (211), and (300) planes were represented by the diffraction peaks that were seen at 2θ values of 21.978°, 31.194°, 38.487°, 50.345°, and 55.510°, respectively. These peaks match well with the hexagonal phase of nickel sulfide,^46^ consistent with the reference pattern PDF#04-013-8923. Additionally, two peaks at 2θ values of 25.630° and 28.817° are attributed to the (100) and (011) planes of cerium sulfide oxide in the hexagonal crystal structure,^47^ as referenced in PDF#04-006-8628. In accordance with PDF#01-087-0712, three additional peaks with 2θ values of 44.496°, 51.849°, and 76.381° correspond to the distinctive diffraction of Ni foam.^48^

The growth mechanism and crystallization process of 2D nanoflakes @Ni foam can be elucidated as follows: when the temperature of the solution media exceeds 90°C, urea decomposes into CO_2_ and NH_3_ gas, which hydrolyzes water to generate hydroxide ions.^49^ The metal salts dissociate into Ni^2+^, Ce^3+^ cations and NO_3_^−^ anions. These cations released from the metal source and OH^−^ from the water form metal hydroxide complexes. Subsequently, the S^2−^ released from the dissociation of sodium sulfide, replaces the hydroxyl ions through an ion exchange reaction, forming metallic sulfide compounds.^22^,^23^ This results in the formation of individual phases of nickel sulfide and cerium sulfide oxide. Cerium hydroxide undergoes partial sulfidation with S^2−^, forming a cerium sulfide oxide phase. The NH_4_F, utilized as a structure-directing agent, also decomposes into NH_4_^+^ and F^−^ anions.^50^ The fluoride ions selectively adsorb on certain crystal facets, directing the anisotropic growth into 2D hexagonal nanoflakes. Nickel foam serves as both a substrate and a supplementary source of Ni^2+^ ions. Its porous structure provides sites for heterogeneous nucleation and growth, thereby facilitating uniform growth of 2D nanoflakes.^51^

Further Raman investigation was carried out on as-synthesized cerium-nickel sulfide 2D hexagonal nanoflakes, as depicted in Figure 2b. The peaks in the Raman spectra are observed at different positions, each corresponding to a vibration of a specific bond in the material. The peak at 143 cm^−^^1^ indicates lattice vibrations within the nickel sulfide crystal structure, contributing to the material’s overall stability. At 243 cm^−^^1^, the observed peak represents the symmetric stretching of Ni-S bonds, demonstrating bond strength and coordination in the hexagonal arrangement. The peak at 299 cm^−^^1^ relates to the asymmetric stretching of Ni-S bonds, while the 348 cm^−^^1^ peak denotes a higher-energy vibrational mode associated with localized Ni-S bond stretching.^52^ The peak observed at 465 cm^−1^ is characteristic of cerium sulfide oxide and is most likely attributed to the lattice structure’s Ce–O stretching vibrations.^38^ These Raman spectra provide crucial information about the hydrothermally produced cerium nickel sulfide nanoflakes. The consistent presence of these peaks aligns with the structural characteristics of the hexagonal cerium/nickel sulfide phase, confirming the successful growth of 2D nanoflakes directly on metallic foam and supporting the XRD observations. The observed Raman peaks in the synthesized 2D hexagonal nanoflakes’ spectra are consistent with existing literature.

Analysis of the adsorption-desorption isotherms shown in Figure 2c reveals that the synthesized 2D nanoflakes on Ni foam exhibit a specific surface area of almost 0.7 m^2^/g. Although the estimated surface area is relatively low compared to two-dimensional materials, this could be due to the high weight contribution of nickel foam. The studied presence of a narrow, intense peak in the pore size distribution (PSD) plot is shown in Figure 2d indicates that the sample is dominated by pores with a diameter of 3.9 nm. Analysis of the PSD graph also shows the presence of pores with larger diameters of up to 10 nm, but their volume is small. The presence of mesopores in the synthesized 2D nanoflakes plays a crucial role in facilitating electrolyte ion diffusion, thereby allowing efficient access of electrolytes and charge carriers to the entire surface of the electroactive material and enhancing energy storage.

**Figure 3:**
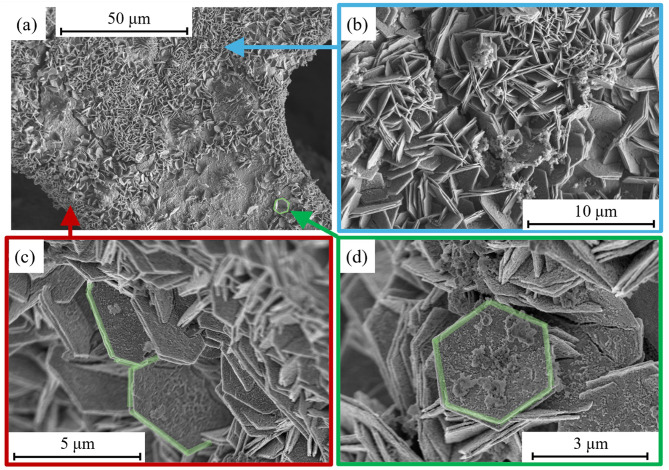
(**a**–**d**) SEM images of 2D nanoflakes @ Ni foam captured at different positions.

**Figure 4:**
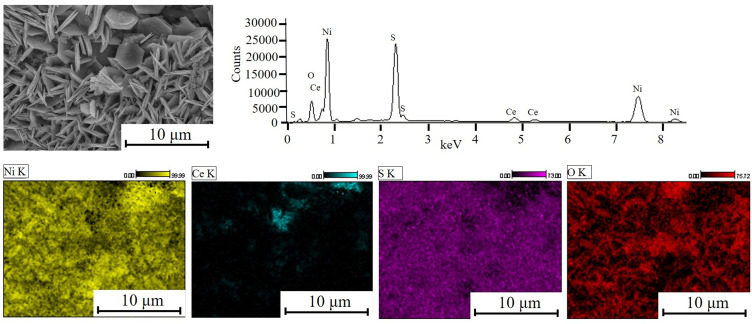
EDX spectrum and maps of element concentration of 2D nanoflakes @ Ni foam.

The SEM images of 2D nanoflakes captured at different magnifications and various positions are shown in Figure 3. It can be seen that these nanoflakes are evenly grown on all sites of the Ni foam. The SEM analysis shows the successful formation of hexagonal nanoflakes with exposed edges, providing a high surface area. The average thickness of the two-dimensional nanoflakes is approximately 60 nm, with an average diagonal diameter of 3.2 μm. Moreover, these nanoflakes exhibit interconnectivity and open channels, facilitating enhanced charge storage capabilities. The elemental composition of 2D hexagonal nanoflakes on Ni foam was confirmed by EDX analysis and is presented in Figure 4. The EDX analysis authenticates the existence of nickel, cerium, sulfur, and oxygen elements. No residual impurities from the precursor were detected, verifying the sample’s high level of purity.

**Figure 5:**
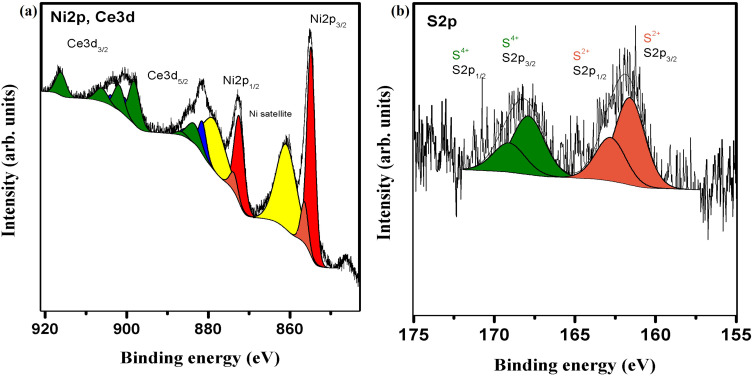
The high-resolution XPS spectra of in situ grown 2D nanoflakes @ Ni foam. (**a**) for Ni2p and Ce3d regions, (**b**) for S2p region.

An X-ray photoemission spectroscopy (XPS) examination was carried out to ascertain the precise chemical composition and states of the constituent components. The high-resolution XPS spectrum recorded for the Ni2p and Ce3d regions is presented in Figure 5a. The binding energies of Ce3d_5/2_ and Ni2p_1/2_ photoelectrons overlap, necessitating simultaneous analysis of these two elements, increasing the interpretation’s complexity. The Ce3d spectrum was deconvoluted into ten peaks, forming doublets characteristic of Ce3d_3/2_ and Ce3d_5/2_ electrons.^53–56^ The relative areas of these peaks provide quantitative information about the contributions of Ce^3+^ and Ce^4+^ oxidation states. The analysis indicates that Ce^3+^ constitutes approximately 27% of the total cerium content, while Ce^4+^ accounts for 73%. The Ni2p core level spectrum was deconvoluted into two doublets consistent with Ni^2+^ and Ni^3+^ species and two additional satellite peaks.^24^,^57^,^58^ The relative contributions of these components were determined to be 85% for Ni^2+^ and 15% for Ni^3+^. The S2p spectrum, depicted in Figure 5b exhibits two distinct peaks relating to the S2p_1/2_ and S2p_3/2_ spin-orbit doublet.^59^ Detailed analysis revealed that the recorded spectrum can be deconvoluted into two doublets, representing approximately 58% of S^2+^ and 42% of S^4+^ species. These components can be interpreted as indicative of sulfur-metal bonds (S^2+^) and sulfur dioxide (S^4+^).^60^,^61^

### Electrochemical Analysis

#### Three-Electrode Setup

**Figure 6:**
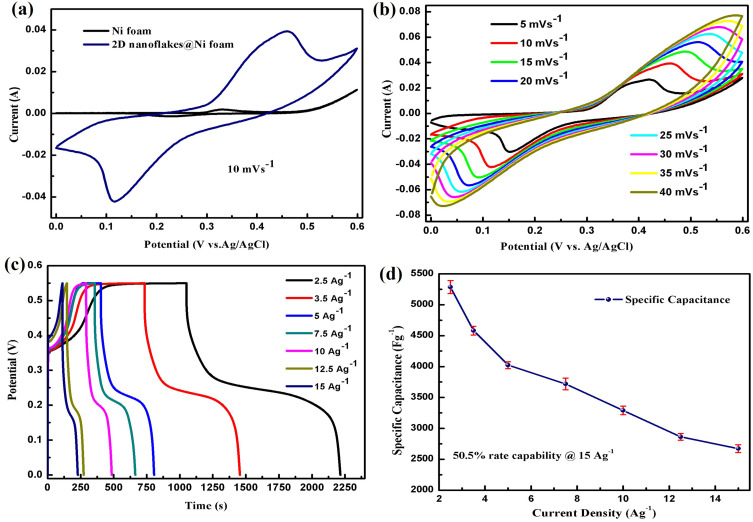
(**a**) CV curves of bare Ni foam and 2D nanoflakes @ Ni foam (**b**) CV curves of 2D nanoflakes @ Ni foam at different scan rates (**c**) GCD curves of 2D nanoflakes @ Ni foam (**d**) capacitance vs discharge current density.

The comparative CV curves at 10 mVs^−1^ for the Ni foam and 2D nanoflakes grown on the Ni foam are presented in Figure 6a. As the enclosed CV curve area is directly proportional to capacitance, it can be inferred from the comparative CV curves that the Ni foam exhibits the least integral area compared to 2D nanoflakes on Ni foam. The influence of capacitance from the Ni foam can be considered insignificant.^62^ Furthermore, CV analysis was conducted on 2D nanoflakes on Ni foam electrode to investigate the redox potential at various scan rates ranging from 5 mVs^−1^ to 40 mVs^−1^ within the potential window of 0–0.6V, as illustrated in Figure 6b. At varying scan speeds, the CV curves clearly show redox peaks that correspond to reversible Faradaic reactions between the electrode and electrolyte. Furthermore, even at greater scan speeds, the redox peaks of 2D nanoflakes are well retained.

The galvanostatic charge/discharge (GCD) technique was employed to examine the charge storage mechanism and to determine the rate capability of the as-synthesized 2D hexagonal nanoflakes on Ni foam. The GCD curves clearly exhibit charge and discharge plateaus associated with reversible Faradaic redox reactions, as depicted in Figure 6c. The rate capability of the prepared electrode was assessed by charging and discharging at various current densities ranging from 2.5A g^−1^ to 15A g^−1^ within the potential window of 0.55V. Figure 6d. Illustrates the relationship between the calculated capacitance using equation (1) and discharge current density. The 2D nanoflakes directly grown on the Ni foam demonstrated remarkable capacitance values of 5286 F g^−1^ at a current density of 2.5A g^−1^ and 2672 F g^−1^ at a current density of 15A g^−1^. Notably, the electrode maintained a 50.5% rate capability even at a higher current density of 15A g^−1^. This high capacitance and rate capability percentage is attributed to the high surface area of 2D nanoflakes, synergistic effects of cerium and nickel-based compounds, and electrode utilization without any binders. The half-cell’s energy density is estimated to be approximately 222.09 Wh/kg with a power density of 687.19 W/kg at a current density of 2.5 A g^−1^.

The CV data were evaluated to determine and quantify the charge storage mechanisms in 2D nanoflakes on Ni foam electrodes. 2D nanoflakes on Ni foam exhibited prominent redox peaks in its CV, particularly at slower scan rates. The presence of peaks in CV curves is an indication of the Faradic charge storage mechanism, which involves reversible redox reactions. In addition, some contribution from non-Faradic processes is observed despite the clear indication of Faradic-based reactions in the redox peaks.^63^ The exact quantification of diffusive and capacitive contribution was carried out using power law, which relates current (i) to scan rate (v) according to Eq. (5).

(5) $$i = a{v^b}$$

According to power law, *a* and *b* are intercept and slope of log of peak current and scan rate. Normally, b value ranges between 0 and 1, which reveals about dominant charge storage mechanism.^64^ A *b* value of 0.5 signifies a purely Faradaic charge storage mechanism, characterized by redox reactions. Conversely, *b* value of 1 indicates a non-Faradaic process. When the *b* value falls between these two limits, it suggests a charge storage mechanism that involves a combination of both Faradaic and non-Faradaic processes.^65^ The exact contribution of capacitive (Q_C_) and diffusive (Q_D_) mechanisms is measured using Dunn’s method from Eq. (6, 7).^66^,^67^

(6) $${{i\left(v \right)} \over {{v^{0.5}}}} = {k_1}{v^{0.5}} + {k_2}$$

(7) $${Q_T} = {\mathrm{ }}{Q_C} + {Q_D}$$

Where, k_1_ and k_2_ are the slope and intercept of plot, respectively. Furthermore, Q_T_ is total charge stored which is combination of capacitive and diffusive charge storage.

**Figure 7:**
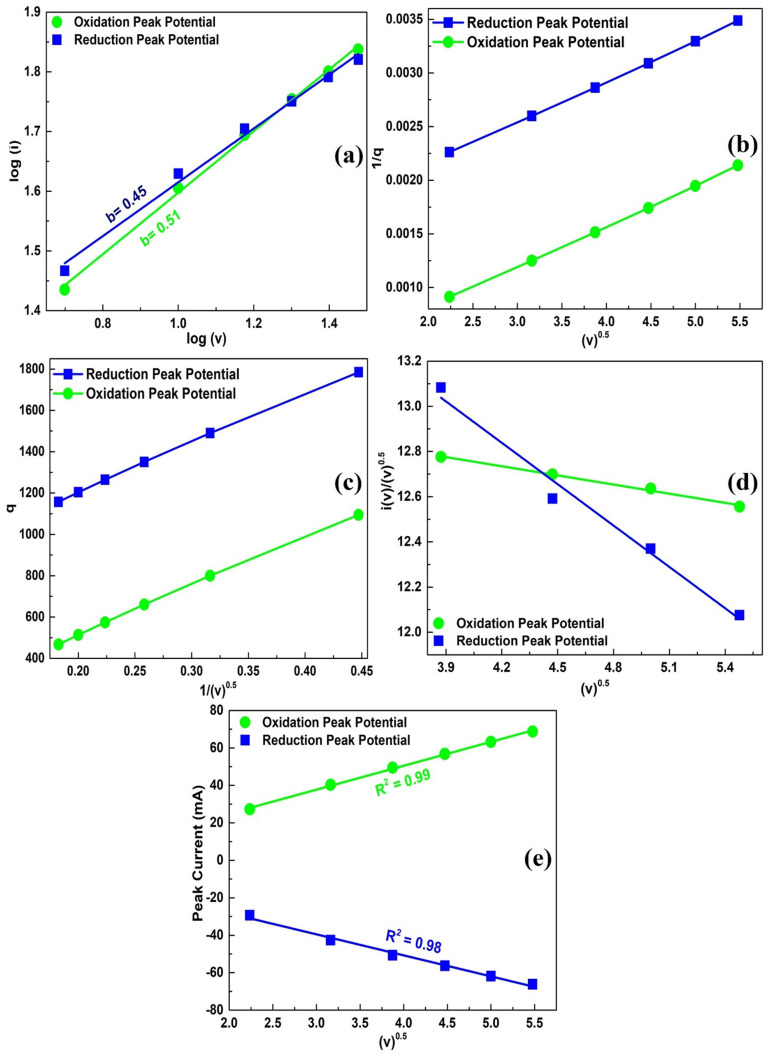
(**a**) b-values of 2D nanoflakes @ Ni foam electrode (**b**–**e**) Dunn’s method plots for calculation of charge storage contribution from CV data at oxidation and reduction peak potentials.

Power law and Dunn’s method plots along respective b values of 2D nanoflakes on Ni foam at oxidation and reduction peak potentials are depicted in Figure 7a–d. The *b* values at oxidation and reduction potentials are 0.51 and 0.45, respectively. *B* values close to 0.5 confirm that the predominant charge storage mechanism is Faradaic in nature, relying on redox reactions for charge storage. The dominance of a diffusion-controlled charge storage mechanism indicates that electrolyte ions are not solely confined to surface-active sites but also diffuse into less accessible active sites. This diffusion process is primarily responsible for the electrode’s high C_sp._^65^

**Figure 8:**
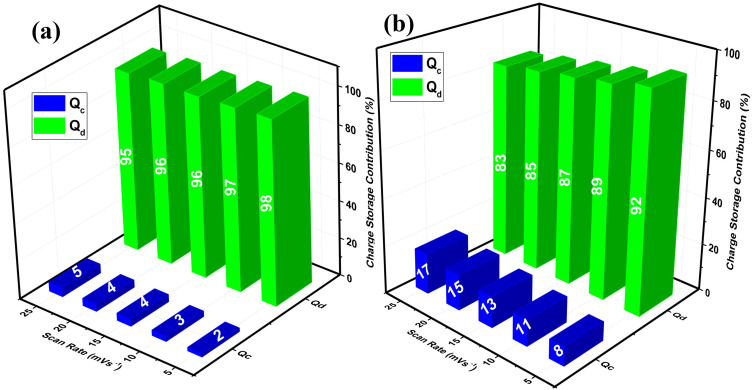
Percentage charge storage contribution calculated at (**a**) oxidation and (**b**) reduction peak potentials and at various scan rates.

Furthermore, Dunn’s method was applied to quantitatively evaluate the evolution of the charge storage mechanism at various scan rates, as shown in (Figure 8a and b). This approach differentiates the relative contributions of capacitive (surface-controlled) and diffusive (ion diffusion-controlled) processes to the overall current response. The bar chart clearly illustrates a systematic variation in these contributions with increasing scan rate. At lower scan rates, particularly at 5 mVs^−1^, the diffusive process predominates, indicating that charge storage is mainly governed by ion diffusion within the bulk of the active material. This behavior arises from the longer time available for electrolyte ions to penetrate deeper into the electrode, facilitating extensive redox reactions at internal active sites.

With increasing scan rate, the capacitive contribution gradually becomes more dominant, while the diffusive component diminishes. This transition reflects a shift from a diffusion-controlled to a surface-controlled charge storage mechanism, as higher scan rates restrict ion intercalation and favor rapid surface adsorption/desorption processes. The observed scan rate dependence of the charge storage behavior thus underscores the excellent kinetic adaptability of the 2D nanostructure, highlighting its efficient ion and electron transport pathways that support both surface and bulk electrochemical activity.

#### Two-Electrode Setup (Asymmetric Supercapacitor Device)

**Figure 9:**
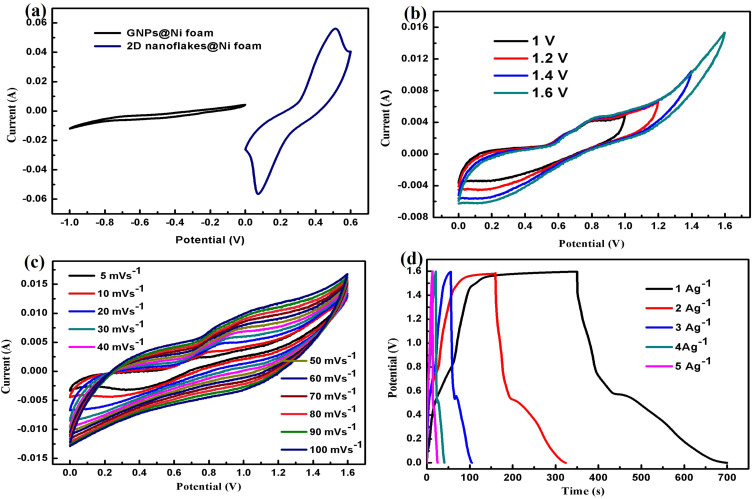
(**a**) CV curves of positive and negative electrodes (**b**) CV curves at the different potential windows (**c**) CV curves of 2D nanoflakes @Ni foam//GNPs@Ni foam (**d**) GCD curves of 2D nanoflakes @Ni foam//GNPs@Ni foam at different current densities.

The charge storage mechanisms of the 2D nanoflakes @ Ni foam and GNPs @Ni foam used as positive and negative electrodes in the asymmetric supercapacitor design were assessed using electrochemical studies. A 2M KOH solution was used as the electrolyte, and the positive and negative electrodes were submerged in it and separated by Whatman filter paper. The developed ASC device’s practical viability was evaluated using CV, GCD, and electrochemical impedance spectroscopy. As shown in Figure 9a, the CV curves performed at 20 mVs^−1^ were merged for both positive and negative electrodes. Figure 9b demonstrates the CV curves performed at 20mVs^−1^ at the potential range of 1–1.6V. The optimal operational voltage range for the asymmetric device was between 0 and 1.6 volts. The CV technique was further conducted for asymmetric supercapacitor between the potential window of 0–1.6V at different scan rates ranging from 5mVs^−1^ to 100mVs^−1^, as depicted in Figure 9c. It was observed that as the scan rate increases, the anodic and cathodic response currents increase. Furthermore, the ASC CV curves combine both charge storage mechanisms: EDLC-type contribution from GNPs @Ni foam and Faradic-type from 2D nanoflakes @Ni foam. Significantly, the ASC device demonstrated steady symmetrical curves with a near-rectangular shape, even when exposed to high scan rates of 100 mVs^−1^. Furthermore, the GCD tests were conducted on the ASC at different current densities from 1 Ag^−1^ to 5 Ag^−1^ within the potential window of 1.6 V, as illustrated in Figure 9d. The capacitance value for the asymmetric supercapacitor was 218 Fg^−1^ at a current density of 1Ag^−1^. The ASC achieved a high energy density of 77.51 Wh/kg and a power density of 797.25 W/kg at a current density of 1 Ag^−1^.

**Figure 10:**
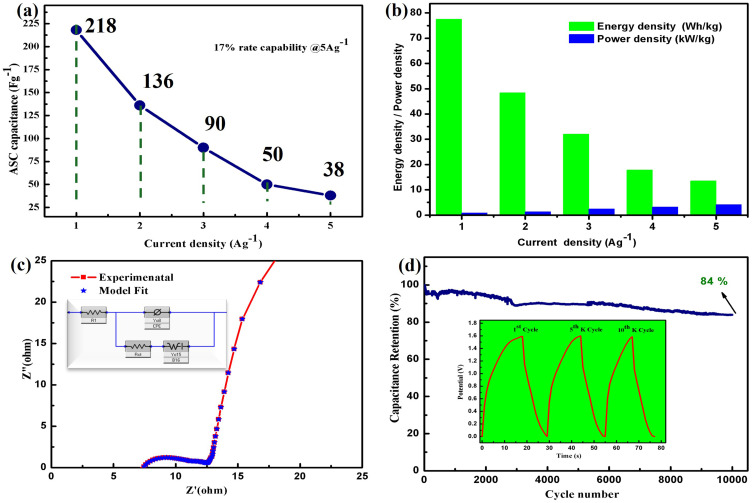
(**a**) Capacitance of ASC 2D nanoflakes @Ni foam//GNPs@Ni foam vs current density (**b**) Energy density and power density of ASC at different current density (**c**) Nuquist plot for 2D nanoflakes @Ni foam//GNPs@Ni foam (**d**) Cyclic performance of ASC 2D nanoflakes @Ni foam//GNPs@Ni foam.

The rate capability of the 2D nanoflakes @Ni foam//GNPs@Ni foam asymmetric capacitor was determined by charging and discharging at current densities varying from 1 to 5Ag^−1^ within a potential window of 1.6V. Figure 10a. Illustrates the calculated capacitance values as a function of current densities. The ASC retained 17% of its capacitance at a current density of 5Ag^−1^. The bar chart in Figure 10b shows the energy density and power density calculated at different current densities of the asymmetric supercapacitor. The ASC demonstrated a high energy density of 77.51 Wh/kg and a power density of 797.25 W/kg at a current density of 1 A/g. At a current density of 5 A/g, the energy density and power density were determined to be 13.51 Wh/kg and 4053.33 W/kg, respectively.

Electrochemical impedance spectroscopy (EIS) measurements were performed using a 10 mV AC perturbation in a two-electrode configuration across the frequency range of 0.01 Hz to 10 kHz in order to further evaluate the electrochemical properties of the asymmetric supercapacitor device. The resultant Nyquist plot shows a linear section at lower frequencies and a semicircular form at higher frequencies, as shown in Figure 10c. As shown in the figure’s inset, the electrical equivalent circuit of the device was ascertained by analyzing EIS data using Echem Analyst software. Warburg resistance, a constant phase element, charge transfer resistance (Rct), and series resistance (Rs) make up this circuit. Based on the semicircle diameter in the mid-frequency band, Rct of the asymmetric supercapacitor was found to be 11.20 Ω. Rct is a measure of the resistance that prevents charge carriers from moving freely at the interface between the electrode and electrolyte during charge transfer. For the asymmetric supercapacitor, the series resistance (Rs) was 7.09 Ω in the high-frequency area of the EIS plot, where the semicircle crosses the real axis. *Rs* encompasses the inherent resistance of the active material, the ionic conductivity of the electrolyte, and the resistance of the current collector. In the low-frequency region of the EIS plot, the linear portion following the semicircle is indicative of ion diffusion dynamics within the electrode-electrolyte system. This region provides insight into Warburg resistance, which reflects ion diffusion behavior. Additionally, the asymmetric supercapacitor was subjected to cyclic performance analysis at a current density of 5 A g^−^^1^. To assess its suitability for practical applications, the device underwent continuous charge/discharge testing for 10,000 cycles. The asymmetric supercapacitor exhibited outstanding cyclic stability, retaining approximately 84% of its initial capacitance, as illustrated in Figure 10d. The ASC’s superior electrochemical performance can be attributed to the binder-free electrode 2D hexagonal nanoflakes @Ni foam utilized as the positive electrode and the high specific surface area provided by the GNPs @Ni foam employed as the negative electrode.

## Conclusion

A quick and effective one-step with microwave assistance hydrothermal method was adopted in this study to directly produce two-dimensional (2D) hexagonal nanoflakes on nickel foam substrates. Direct in-situ development of 2D nanoflakes on the conductive substrate was made possible by the moderate reaction environment produced by the combination of microwave heating, hydrolysis agent urea, and morphology-directing additive ammonium fluoride. The nanoflakes were found to have a diagonal dimension of around 3.2 μm and an average width of about 60 nm, according to SEM research. XRD was used for structural analysis, which validated the 2D nanoflakes’ hexagonal crystalline structure, and Raman spectroscopy confirmed the bonding properties.

The nanoflake-coated nickel foam electrodes demonstrated remarkable electrochemical characteristics, such as enhanced capacitance and outstanding rate capability. Due in large part to its distinctive morphology, the electrode demonstrated a remarkable specific capacitance of 5286 F/g, energy density of around 222.09 Wh/kg, and power density of 687.19 W/kg at a current density of 2.5 A/g when tested in a half-cell arrangement. The enhanced electrochemical performance of the Ni_3_S_2_/Ce_2_O_2_S 2D nanoflake electrode can be attributed to several synergistic structural and compositional factors. The unique hexagonal 2D morphology offers a large surface-to-volume ratio, facilitating rapid ion diffusion and enhancing electrolyte accessibility to electroactive sites. Furthermore, the coexistence of Ni_3_S_2_ and Ce_2_O_2_S phases forms a heterostructured interface that promotes efficient electron transport and multiple redox transitions through synergistic interactions. Additionally, at 1 A/g, an asymmetric supercapacitor using graphene nanoplatelets (GNPs)@Ni foam as the negative electrode and 2D nanoflakes@Ni foam as the positive electrode produced a power density of 797.25 W/kg and an energy density of 77.51 Wh/kg. Additionally, the ASC showed outstanding cycle stability, holding onto around 84% of its original capacitance even after extended cycling. This work stipulates imperative understandings into the design of a sophisticated binder-less electrode for next-generation energy storage technologies, as it represents the first successful synthesis of hexagonally structured cerium/nickel sulfide 2D nanoflakes.
